# Non-physician providers of obstetric care in Mexico: Perspectives of physicians, obstetric nurses and professional midwives

**DOI:** 10.1186/1478-4491-10-6

**Published:** 2012-04-25

**Authors:** Lisa M DeMaria, Lourdes Campero, Marianne Vidler, Dilys Walker

**Affiliations:** 1 Center for Health Systems Research, Instituto Nacional de Salud Pública, Cuernavaca, Mexico; 2 Center for Population Health Research, Instituto Nacional de Salud Pública, Cuernavaca, Mexico; 3 School of Public Health, Simon Fraser University, Vancouver, BC, Canada; 4 School of Public Health, University of Washington, Seattle, WA, USA

**Keywords:** Midwifery, Obstetric care, Mexico, Obstetric nurses, Skilled birth attendants

## Abstract

**Background:**

In Mexico 87% of births are attended by physicians. However, the decline in the national maternal mortality rate has been slower than expected. The Mexican Ministry of Health’s 2009 strategy to reduce maternal mortality gives a role to two non-physician models that meet criteria for skilled attendants: obstetric nurses and professional midwives. This study compares and contrasts these two provider types with the medical model, analyzing perspectives on their respective training, scope of practice, and also their perception and/or experiences with integration into the public system as skilled birth attendants.

**Methodology:**

This paper synthesizes qualitative research that was obtained as a component of the quantitative and qualitative study that evaluated three models of obstetric care: professional midwives (PM), obstetric nurses (ON) and general physicians (GP). A total of 27 individual interviews using a semi-structured guide were carried out with PMs, ONs, GPs and specialists. Interviews were transcribed following the principles of grounded theory, codes and categories were created as they emerged from the data. We analyzed data in ATLAS.ti.

**Results:**

All provider types interviewed expressed confidence in their professional training and acknowledge that both professional midwives and obstetric nurses have the necessary skills and knowledge to care for women during normal pregnancy and childbirth. The three types of providers recognize limits to their practice, namely in the area of managing complications.

We found differences in how each type of practitioner perceived the concept and process of birth and their role in this process. The barriers to incorporation as a model to attend birth faced by PMs and ONs are at the individual, hospital and system level. GPs question their ability and training to handle deliveries, in particular those that become complicated, and the professional midwifery model particularly as it relates to a clinical setting, is also questioned.

**Conclusions:**

Hospitals in the Mexican public health sector have a heavy obstetric workload; physicians carry the additional burden of non-obstetric cases. The incorporation of a non- physician model at the primary health center level to attend low-risk, normal deliveries would contribute to the reduction of non-necessary referrals. There is also a role for these providers at the hospital level.

## Background

Ensuring skilled attendance at birth is widely acknowledged to be a critical factor to ensuring successful birth outcomes for both the mother and baby [1,2]. Indeed, the authors of the recent report documenting the decline in maternal mortality rates over the past twenty years partly attribute this change to increased skilled attendance at birth [3]. In health sectors worldwide, increasing attention is being paid to the incorporation of skilled alternative providers into the health system as a key strategy to reduce maternal and neonatal mortality rates [4]. In the case of many middle-income countries, such as Mexico, this means broadening the focus from a purely physician-centered model of labor and delivery care to include other, non-physician providers who also meet the criteria of skilled birth attendants. The broadened focus on access to skilled attendance at birth, instead of a physician-centered model, together with growing emphasis on midwifery training as a specific human resource prepared to meet women’s needs at birth, has resulted in a renewed commitment to not only focus on improved quality of medical care but also on assuring humanized birth, respecting the opinions and preferences of women throughout the birth process.

During the 1960s, with the aim of reducing maternal mortality and morbidity, the Mexican government implemented policies that exclusively promoted physician attendance at delivery as well as institutionalized delivery, sending a clear message that a safe delivery is one attended by a physician. This strategy firmly placed obstetric care in the hands of physicians. As a corollary, the profession and role of the midwife and obstetric nurse working independently were eliminated in the public health system. Over the last half century the proportion of births attended by physicians has increased nationally from 55% in 1974 to 87% in 2003 [5,6]. General practice or family care physicians attend the majority of these births and specialized care by OB/GYNs is concentrated principally in the larger urban areas. However, despite the promotion of institutional delivery, the decline in the national maternal mortality rate has been slower than expected. In part this slow decline may be due to the fact that in many rural areas *pasantes*—medical students in their final year of training completing their social service requirement—are providing the bulk of the care. These *pasantes* often have limited obstetric training, and do not stay in the communities to which they are posted beyond the 12-month commitment. Other problems plaguing the delivery of obstetrical services in the Mexican health system include saturation of secondary and tertiary hospitals with routine, normal deliveries, high turnover of physicians in rural areas, disrupting the continuity of care, and a rural population that bears the brunt of the burden of maternal mortality. Non-physician providers, primarily professional midwives, have been proposed as a strategy to ameliorate these problems.

Recently, the Mexican Ministry of Health has published a maternal health strategy that highlights the need for alternative skilled providers and increased emphasis on humanized birth [7]. In Mexico, there are two non-physician models that meet criteria for *skilled attendants* as defined by International Confederation of Midwives and adopted by the WHO [8,9]: professional midwives and obstetric nurses [10]. There are limited midwifery training programs and opportunities in Mexico, which vary in terms of requirements and structure. Currently CASA Midwifery School in San Miguel de Allende is the only school in Mexico to train midwives and confer a technical license for its graduates after completing 3 years post-secondary school training. Obstetric nurses have a 4-year university level nursing degree that includes one year of obstetric training. The National School for Nursing and Obstetrics (ENEO), part of the National Autonomous University of Mexico in Mexico City, is the main school for obstetric nurses, and a handful of other schools exist around the country. Medical schools are more prolific; every state in Mexico has at least one Faculty of Medicine.

Both obstetric nurses and professional midwives are underutilized by the health system in Mexico and attend only a small fraction of the births in specialized settings. Midwifery is not a recognized profession, and only in 2011 has a budget code been introduced, allowing them to be contracted by the health system. Obstetric nurses are similarly underutilized and despite the re-instatement of their professional budget code in 2008, few have been contracted.

Since 2005, a group of researchers at the National Institute of Public Health in Mexico have been working to build the evidence base regarding the use of these non-physician labor and delivery care providers. A curriculum review [10] followed by a transversal observational study of professional midwives, obstetric nurses and general practice physicians demonstrated that professional midwives and obstetric nurses perform at the same or superior levels as their general physician counterparts in the provision of labor and delivery care [11]. However, it has been observed that these providers face structural and individual barriers to their greater integration into the Mexican health system.

In order to more fully understand the potential contribution of these providers to fulfilling the Ministry of Health strategy to increase the role of non-physician providers as well as the barriers to their integration into the health system, this article presents results from a qualitative study of the same three types of providers.

The objective of this study was to compare and contrast these three types of provider’s perspectives on their respective training, scope of practice, and also their perception and/or experiences with integration into the public system as skilled birth attendants.

## Methods

The present paper is derived from and synthesizes qualitative research that was obtained as a component of the quantitative and qualitative study that evaluated three models of obstetric care: professional midwives (PM), obstetric nurses (ON) and general physicians (GP).

This cross-sectional study was conducted among providers in five hospitals with a range of models for obstetric care but with similar basic characteristics in that the majority of the births are attended by non-specialists. Specialty consultation and cesarean capacity varied among the hospitals, with only one site unable to perform cesarean sections. Four hospitals had specialized physicians on call or present part time; one site had to refer all women requiring specialized care to a hospital one hour away by car. Three of the hospitals were government-run public hospitals (Teocelo, Veracruz State; Aquismon, San Luis Potosi State; and Chilapa, Guerrero State) and two hospitals were run by non-governmental organizations (CASA in San Miguel de Allende, Guanajuato; and CimiGEN in Mexico City). Each hospital offers a different model for obstetric care. The “pure models” were: Chilapa (allopathic medical model); CASA (midwifery); and CimiGEN (obstetric nurse). Two were “mixed models”: Aquismon (medical and midwifery) and Teocelo (medical and obstetric nurses) (See Table 1).

**Table 1:** Sites and types of providers interviewed

**Site name(type of hospital)**	**Model of obstetric care**	**Provider type**			
		**ON**	**PM**	**Physician**	
				**GP**	**Spec.**
Cimigen (private NGO hospital)	Obstetric Nurse	3 F + 1 F pasante	0	0	1 M,1 F
CASA (private NGO hospital)	Midwifery	0	4WF + 2 F SS	1 M	0
Teocelo (government hospital)	Mixed: obstetric nurse and medical	4 F	0	2 M, 2 F	0
Aquismon (government hospital)	Mixed: medical and midwifery	0	1 W	2 M	0
Chilapa (government hospital)	Medical	0	0	1 F	1 M

Abbreviations: *ON* obstetric nurse, *PM* professional midwife, *GP* general physician, *Spec.* specialist (OB/GYN or surgeon), *F* female, *M* male.

The data collection was conducted in two steps. The first phase consisted of 17 semi-structured individual interviews with each type of provider between July and August 2006 (2 PM, 6 ON, 3 OB/GYN, 6 GP) conducted by a trained qualitative interviewer, lasting between 1 and 1.5 hours. Ten interviewees were female, two were male specialists and 5 male general physicians. The second phase of the study consisted of follow-up interviews to expand on the results and opinions presented in the interviews of the first phase. These interviews took place in two of the hospitals (Teocelo and CASA) during March and April 2007. In this phase, ten interviews were conducted with various providers: five professional midwives (all female graduates of CASA), two female obstetric nurses, and three general practitioners (2 male and 1 female). The objective of these interviews was to provide a clear description of their practice and model of care, perceptions of other providers, and their interaction with the health care system.

Interviews were transcribed following the principles of grounded theory [12], codes and categories were created as they emerged from the data. With the support of ATLAS.ti we analyzed data. Through the testimonies, several themes were identified, among them: training for obstetric care; professional trajectory; attention/care during labor and delivery; perceptions of women’s emotional states during labor; abortion; limitations of practice; perceived advantages and disadvantages of each provider; the transformation of the practice; institutional and interdisciplinary barriers; and maternal mortality. However, for this paper, we prioritize those themes related to our mentioned objective, organized around four topics: I. Training and limits of practice; II. Variations in practice; III. Perceptions of other models of care; and IV. Perceptions regarding integration of non-physician providers into the health system.

Prior to the initiation of this study, the protocol and instruments were approved by the Ethics and Research Committees of the National Institute of Public Health.

## Results

The interviews revealed a range of similarities and differences between obstetric care providers. There are clear differences in the training, scope and model of practice among the different provider types, which can lead to professional clashes when providing maternal care together in the same institution. An important element of the results included the perceptions of benefits, and limitations of practice, of providers. Finally, the acceptance and potential incorporation of professional midwives and obstetric nurses into the healthcare system is addressed.

### Provider perspectives on respective training programs: Some strengths and limitations

While a complete overview of the training curriculum for each provider and the differences between them can be found elsewhere [10], it is relevant to examine the comfort levels of providers, comprehensiveness of training and the limitations of practice in the various models of care. To frame this discussion, we selected three specific practices that illustrate their perspectives on autonomy, skills and practice norms: episiotomy, non-pharmacologic pain management and uterine wiping. (Uterine wiping, a practice routinely carried out post-vaginal delivery in Mexican public hospitals, consists of inserting a few fingers or hand covered in gauze, or forceps with gauze, into the uterus after delivery of the placenta, and thoroughly wiping the inside of the cavity to ensure that there is no placental tissue or membranes remaining.)

Training for professional licensed technical midwives is three years followed by one year of social service in a hospital setting. Professional midwives reported feeling confident with respect to their training due to its strong basis in theory and clinical practice. The positive aspects of their training, mentioned by the professional midwives include: interdisciplinary instruction, the ability to work both within the hospital setting and the community, and the focus on managing uncomplicated obstetric cases and identifying risk.

During the social service year, professional midwives expand their practical experience with direct supervision. In this year they must attend at least 20 births, though many attend more. One midwife mentions, “I think the social service is the best complement because we are sent to hospitals where there is a lot of work and there we complete our training.”

Professional midwives also train in the community with traditional birth attendants, providing a unique and important component to their education. One professional midwife reported:

"“In my training, I learned a lot from the traditional midwives… One of these things is their sensitivity, their respect and love towards the women; the trust developed between the woman and her traditional midwife… From this trust emerges the confidence that the labor will go well. If [a woman is] scared, stressed or frightened [she] will not have a normal birth because it inhibits labor.” (Midwife)"

"The training of obstetric nurses includes four years of university level study, one of which is dedicated specifically to obstetrics, and one additional year of social service. As a result of their training, obstetric nurses were confident in identifying risk factors. “I believe that [our training] is good, because they teach us to identify risk factors that could endanger the lives of the women and infants.” (Obstetric Nurse)"

ON training prepares them to focus on managing normal labor and delivery as well as on a supportive role in obstetric care: including preparing the delivery room and instruments, providing non-pharmacologic pain management, assisting the anesthesiologist, assessing indications for episiotomies and suturing the subsequent incision, delivery and postpartum care, and understanding relevant pharmacology. While they are able to identify and refer emergencies to the appropriate provider, these providers emphasized that they are always under the supervision of physicians.

"“They [the specialists] only help us to check to make sure everything is going well, and … help us make notes [in the clinical chart], but apart from that we do everything.” (Obstetric Nurse)"

The course of studies for general practitioners is a six-year degree program: four years of classwork and study, one year of social service and one year as a medical intern. During their schooling there is a 6-week rotation in obstetrics that provides limited and varied practical training. Although practical experience was lacking during the years of schooling, general practitioners felt confident in their abilities to manage maternal care because of the experience during their social service year and internship:

"“Here in this hospital I learned a lot about [gynecology and obstetrics] when I was an intern… I had the good luck of having on a gynecologist who explained everything well to me: what is labor, the care during labor and the pediatrician too [taught me about] neonatal resuscitation.” (Doctor)"

Some general physicians commented that although they have a complete understanding of obstetric theory, their training focuses on the unpredictability of childbirth. General physicians reported that this inherent unpredictability presents challenges in managing emergency cases.

"“Really I feel quite confident in my performance [attending deliveries …]. Anyway there is always a certain level of stress when attending births because it is an unpredictable event; it is always unpredictable, many things can be predicted but not everything. So to be totally confident [in your approach to birth] can be a type of neglect”. (Doctor)"

General practitioners, professional midwives and obstetric nurses are all trained to provide basic emergency obstetric care, but do not perform surgeries such as caesarean sections and cannot repair extensive lacerations. Therefore, they must operate within a clinical framework that includes either referral or on-site specialty care. Respondents from all three models felt capable of attending low-risk situations and indentifying obstetric cases for referral during pregnancy, delivery, or postpartum. The role of specialists varied among the study sites, namely in the extent to which they had a supervisory role in addition to being on call for consults of complicated cases and cesarean sections. However, in all sites, they are critical to a functioning health system with regards to management of complicated cases.

### Variations in practice

We analyzed the variations of practice of the three models of care, with regards in particular to pain management, episiotomy, uterine wiping and patient interaction. The WHO guidelines for care during normal birth clearly states that non-pharmacologic pain control should be promoted, and that episiotomies and uterine wiping are not routinely indicated [13].

Professional midwives are proactive in the management of care of the perineum to avoid episiotomies, which is in accordance with recommendations on safe births [14]. One professional midwife described her approach as one of patience and caring for the perineum:

"“I cover the perineum, I wait for the head to come out perfectly, I wait for the baby to turn around perfectly so it will come out little by little, I wait to cut the cord, I pass (the baby to) the nurse to dry and warm him under the lamp. I wait for the placenta to be expelled.” (Midwife)"

Physicians and obstetric nurses reported greater use of episiotomies in efforts to prevent tears. They decided if an episiotomy was needed based on the direct assessment of the woman during birth or on parity, with nulliparous woman more likely to require an episiotomy as they have a “tight” or “resistant vagina” that will impede a quick birth.Uterine wiping was performed in at least some cases by all providers. Professional midwives performed this only when absolutely necessary, namely when there was a concern of retained placental tissue, and PMs attempted to avoid what they viewed as an invasive and traumatic procedure. In contrast, the majority of obstetric nurses and physicians interviewed reported performing uterine wiping routinely. “As much as you may want that the placenta be complete sometimes it is not… There are many complications that can arise from not doing a uterine cleaning… It should be done.” (Obstetric Nurse)

All professional midwives and obstetric nurses interviewed reported employing a variety of strategies for pain management during labor including massage (reported by nearly all interviewed), aromatherapy, warm baths and walking. A professional midwife explains, “During the active stage of labor is when women start to worry, and we start to massage, use aromatherapy, homeopathic medicine to [help her] relax, and run a hot bath.” In addition to these techniques professional midwives and obstetric nurses used deep breathing exercises and encouraged women to ambulate and change positions to help relieve pain and stress during labor.

General practitioners were much more limited in their approach and alternatives to pain management. While they recognize the utility of non-pharmacologic pain management, they were not trained in techniques. Some mentioned breathing exercises, although more to ensure that the newborn has adequate oxygen levels, than for benefitting the laboring woman. Additionally they face important structural barriers to employing non-pharmacologic pain management techniques. While some doctors recognized the utility of letting a woman walk during labor, structural barriers in the hospital were a limiting factor in encouraging this practice.

"“When the women are admitted into the labor room, they definitely do not walk and they stay in the bed. When the emergency room doctors see that the women have a long way to go and are 2 or 3 cm dilated, they frequently send them home, as long as they live close. If they live far away, they send them outside [of the hospital] to walk”. *(Doctor)*"

The various providers had different approaches to care and interact with their patients. Professional midwives viewed every woman and their experience as unique. Most implemented a passive approach to delivery, avoiding force, and waiting to allow a natural birth. Obstetric nurses and professional midwives reported encouraging relaxation to alleviate fears and tension. General practitioners, especially those working in hospitals that had an important proportion of indigenous populations, mentioned the challenge presented by language barriers with patients, even if a translator was present. General practitioners framed their interaction with the woman in labor in terms of advising her of what to expect, frequently with the aim to having the woman be “cooperative” during her labor and delivery.

"“From the time I see the patient, I tell her: ‘You are going to cooperate because it is like this and like this. What is going to come is going to be stronger and I want you to cooperate, because if you don’t, we are all going to get frustrated, okay?’ It isn’t about screaming or kicking. Don’t try to get all twisted up, but try to cooperate.” *(Doctor)*"

### Provider perspectives of other models of care

The perception of each model varied according to level of exposure to other models of care. As can be expected, among providers at “pure” sites, there was a lack of understanding and appreciation of the competencies and models of care of other types of obstetric providers. Physicians did not have a clear understanding of professional midwives as a profession separate from an untrained traditional birth attendant. Additionally, the traditionally hegemony of physicians in Mexico led to the perception of obstetric nurses in a supporting role to the physician, a perspective that persisted even in the mixed sites.

Among those providers coming from mixed model settings, there was a greater understanding of these other models of care. Those general physicians exposed to the model of licensed midwives find them to be well prepared to manage obstetric patients:

"“In reality I have been working very well with the professional midwife that we have here, and they have some advantages over the doctor. … They have [three (sic)] years of pure obstetrics. None of them have had any medical training, but they had [three (sic)] years of pure obstetric nursing, I say that this is pure attention to deliveries.” (Doctor)"

Yet there was recognition of certain limitations such as structures preventing midwives from prescribing medications, ability to manage complicated cases or work independently:

"“A midwife identifies a problem, because she knows how to. However she needs us to confirm her diagnosis. For instance the midwife says: ‘You know what, it looks like this problem is happening, I need you to evaluate the patient.’ Then it turns into a situation where a someone [a doctor] who was doing something, has to stop what he was doing and go check and see if it is a problem or not.” (Doctor)"

Likewise, physicians with exposure to obstetric nurses perceived them to be competent in assisting with low-risk deliveries:

"“I think that it is great that obstetric nurses are part of the hospital staff; I think that it is really good […], Here we consider them capable and qualified and we believe very much in their work.” (Doctor)"

Professional midwives argued for their attendance of low-risk births based in part on their beliefs that patients prefer female providers. As such, professional midwives perceived themselves to be more empathetic with patients and less costly to the system and reducing the workload of general practitioners. Like professional midwives, obstetric nurses perceived themselves and are perceived by physicians to have a gender advantage allowing for greater empathy and personalized care.

General practitioners believed their education and clinical skills qualify them as the most competent providers. The majority believed their model of care yields superior maternal and neonatal outcomes and in the importance of their role as supervisors. However, professional midwives and obstetric nurses considered the work of physicians to be routine, cold, occasionally aggressive, and lacking in personalized care:

"“We still run into a couple of people who tell to the patient, ‘Open your legs and start pushing. You have done it once, now you can do it again.’ These are rather aggressive things to say, no? Especially in that moment when the woman is much more sensitive to everything, and maybe she’ll never forget what you said […].” (Obstetric Nurse)"

### Provider perspectives of integration of non-physician providers into the health system

Providers, particularly physicians, differed in their support for integrating professional midwives and obstetric nurses into the healthcare system. In general, physicians that were exposed to midwifery or obstetric nursing models of care were more accepting of integration of these models into the health system, yet are pragmatic when considering the barriers to incorporating these non-physician obstetric providers. The primary areas of concern among physicians were qualifications and the potential competition for jobs with graduating physicians. The majority of physicians supported integration of other provider types, provided they are qualified and do not compete directly for physicians jobs.

"“There has been rejection [of these providers] by some physicians here, but I think we need to keep an open mind, and that we should accept this type of provider …I see them as a support. There are a lot of doctors who think of them as competition, but you need to see how much one person can do, and see the help that they provide.” (Doctor)"

"“There is professional jealousy among the doctors, and they do not accept that a nurse can attend a delivery.” (Doctor)"

Professional midwives and obstetric nurses also supported the idea of opening positions for them to attend low-risk pregnancies and births. However, these non-physician providers expressed concern as differences in standards of practice regarding obstetric management among the different provider types have occurred, especially between professional midwives and doctors, in regards to the use of non-sterile fields, attending deliveries in beds and not in a specific delivery room, and the PM resistance to performing routine episiotomies and uterine wiping.

Conflict between physicians and obstetric nurses has also been observed; a professional jealousy has precipitated between the two particularly when the capabilities of obstetric nurses are not recognized. For example, obstetric nurses in one site reported friction when their specific skill set was not recognized where general nursing duties such as washing and exchanging material, taking vital signs and doing administrative work, were assigned to them.

Most physicians thought it would be easier to incorporate obstetric nurses into the health system than professional midwives. Obstetric nurses were accustomed to working in hospitals and there are more similarities in the models of care.

"“I am not against contracting professional midwives or obstetric nurses. However, I think that the type of training that PMs go through is oriented to attending patients outside of health clinics and hospitals because it is a more personalized, community-oriented approach. We see them interacting with the traditional midwives and working with them.” (Doctor)"

## Discussion

The Mexican Ministry of Health has adopted the strategy of incorporating non-physician providers into the health system for the provision of obstetric care, but has limited data to inform the implementation process for this policy at the operative level. Recent quantitative evidence shows that Mexican-trained midwives and obstetric nurses perform at a similar level or even above that of general physicians in process indicators of obstetric care and their patients have similar neonatal and obstetric outcomes [10,11]. Yet, incorporating these non-physician providers presents challenges to the health system in terms of resistance by current health provider cadres and managers, frequently due to unfamiliarity with these other provider types, lack of clarity regarding optimizing human resources at the primary, secondary and tertiary levels, and differences in approaches to care.

All provider types interviewed expressed confidence in their professional training and acknowledge that both professional midwives and obstetric nurses have the necessary skills and knowledge to care for women during normal pregnancy and childbirth, each with different technical strengths and characteristics. The three types of providers recognize limits to their practice, namely in the area of managing complications.

We detected differences in how each type of practitioner perceived the concept and process of birth and their role in the process. Professional midwives clearly prioritized humanistic care during childbirth and aimed at creating a natural birth experience. The model of care provided by obstetric nurses lays somewhere in between: warm treatment and respectful care is emphasized at all times, considering the patient wishes regarding childbirth, yet also placing importance on the clinical procedures and hospital protocols. They have a similar clinical perspective to physicians, and therefore have an easier time when communicating with them. GPs viewed the birth process as something to be controlled where something may go wrong at any time; they did not appreciate the autonomy and rights of the laboring women, a perspective documented elsewhere [15]. They also had the limitation of being trained and working within a system that reinforced this view, making adopting a different perspective even more difficult. These varied perspectives were clearly illustrated by their different approaches to obstetric practices such as conducting episiotomies, pain management and uterine wiping, as well as their interaction with the patients. PM and ON training specifically incorporates perspectives and practices which fosters an approach that respects the rights and autonomy of the woman and aims to create a respectful environment for their birth; these competencies and concepts are lacking in the physician training. It is time for women’s autonomy and respectful care to be prioritized, particularly in poor, rural communities. The midwifery model seems the most likely to achieve this goal.

The barriers faced by professional midwives and obstetric nurses are at the individual, hospital and system level: general physicians question their ability and training to handle deliveries, in particular those that become complicated, and the midwifery model particularly as it relates to a clinical setting, is also questioned. The slower-paced, humanistic model of birth is not always seen as feasible in a busy hospital setting. Nonetheless, managing patient autonomy and being respectful is always of upmost priority and does not take any additional time on the part of the provider.

There is debate at the national level regarding integrating midwives into primary, secondary and tertiary levels. While some admit that there may be a role for midwives at the hospital level, some providers (obstetric nurses in particular) believe that given the emphasis on integration at the community-level during the midwives’ training, a better fit would be at the primary health care level. The scarcity of jobs for either provider, and for physicians, likely contributes to this friction about the appropriate place and roles for the non-physician providers.

Many of these barriers arise from physicians’ limited exposure to these other models of care. All midwives and obstetric nurses who had experience working in a medical setting have had to work to prove their capacity to the physician colleagues and to protect their model of care. After having the time to observe these other providers, physicians have come to understand and respect their models of care. In particular, midwives must work even harder to differentiate themselves (in the eyes of the physician) from the traditional midwife, while respecting the training by this provider. The tension within the existing hierarchies of medical and non-physician providers has been observed in many other countries as well [16-18].

Finally, there is the opportunity to significantly impact quality of care by introducing PMs and ONs at the primary care level. Currently, rural, primary care clinics depend on the work of *pasantes* during their social service year and those who have recently completed their medical studies. This strategy automatically challenges continuity of care, as none of these young physicians stay at their site for more than 12 months. Additionally, these young doctors receive very little supervision and are expected to independently attend deliveries and identify obstetric complications with their limited experience. Incorporating PMs in particular into these health centers has clear benefits. Many PMs come from rural, indigenous areas and are keen to return to their communities. Additionally this cadre receives training on working at the community level and is skilled at community outreach and navigating the cultural aspects of working in rural areas.

The main limitation in the design of this study is that there are few hospital-level settings in Mexico where non-physician providers are practicing and providing obstetric services. As a result, we resorted to a convenience sample and findings may not be representative, in particular as related to the physicians. We did include the main practice training sites for midwives and obstetric nurses for this study. However, in 2010, in Atlacomulco, in the state of Mexico, a maternity hospital was inaugurated that is staffed entirely by obstetric nurses and peri-natal nurse specialists (obstetric nurses with an additional year of specialty training). Any complicated cases or those requiring cesarean section are referred to the local general hospital [19]. This effort is to be commended and deserves close monitoring and evaluation. As with any new health policy, the results of incorporating ONs and PMs into the health care system should be closely monitored and evaluated to estimate the impact on maternal and neonatal outcomes as well as make adjustments to the policy to ensure that this strategy optimizes care.

## Conclusion

Hospitals in the Mexican public health sector already have a heavy obstetric workload; physicians carry the additional burden of non-obstetric cases. This obstetric burden is not expected to abate soon and it is increasingly common for hospitals to receive referrals even for low-risk labor and deliveries. To have skilled providers at the primary health center level with the confidence to attend low-risk, uncomplicated deliveries would surely contribute to the reduction of non-necessary referrals. Even in busy referral hospitals complementary and mutually supportive roles for physicians, professional midwives, and obstetric nurses can be envisioned. While this study focused primarily on issues related to care during labor and delivery, it is clear that there is a role for these alternative providers as primary care providers in the provision of prenatal and post partum care as well.

The administrative aspects of incorporating these providers should not be overlooked. In February 2011, the professional midwives were assigned a code in the health administration system, thus allowing contracts for this type of personnel [20]. However, there still exists resistance to employing non-physician professionals for obstetric care at the various levels of the health system, and guidelines as to how to fully incorporate these provider types do not exist. Also some degree of inter-professional antagonism and competition is inevitable if PMs and ONs were to be further integrated with a more independent role for providing care during labor and delivery.

Obstetric nurses, professional midwives, general practitioners and specialists have very different training; however, there is a place for each provider type in an integrated, multidisciplinary model of care. Collaboration between care providers will be enhanced through a greater understanding and respect of roles, competencies and perspectives of care. The Ministry of Health has an important role in implementing policies and regulations to allow for contracting of midwives and obstetric nurses, as well as promoting the evidence supporting the use of these non-physician providers of obstetric care in a hospital setting.

## Competing interests

The authors declare that they do not have any competing interests.

## Authors’ contributions

LC, LD, DW designed the study instruments. LC supervised the data collection and codification process and carried out the preliminary analysis. LD, LC, MV and DW conducted the analysis and participated in drafting the final article. All authors read and approved the final manuscript.
